# Sexual function after surgically corrected menstrual outflow obstruction due to congenital anomalies

**DOI:** 10.52054/FVVO.16.4.049

**Published:** 2024-12-27

**Authors:** L Martens, M.A. Spath, M.A. van Beek, W.N.P. Willemsen, K.B. Kluivers

**Affiliations:** Radboud University Medical Center, Radboud Institute for Health Sciences, Department of Obstetrics and Gynecology, Nijmegen, The Netherlands; Present work address: Care group Solis, Deventer, The Netherlands

**Keywords:** Congenital anomaly, menstrual obstruction, Müllerian anomaly, reconstructive surgery, sexual function, questionnaire

## Abstract

**Objectives:**

To enhance evidence-based knowledge about sexual function and the prevalence of sexual dysfunction after surgical therapy for congenital anomalies with menstrual outflow obstruction.

**Materials and Methods:**

In this long-term case-control study, all patients underwent surgical correction of an obstructive Müllerian anomaly between 1980 and 2013. At the start of the case-control study, patients were at least 18 years old and were two years post their initial operation. The control group were women without current gynaecological problems. 38 patients (response rate 48.7%) and 54 controls were included. Chi-square test linear-by-linear Association, Fisher’s Exact Test, Mann-Whitney U test and the unpaired sample t-test were used for statistical analysis.

**Main outcome measures:**

The following questionnaires were used: the Female Sexual Function Index (FSFI), the Body Exposure during Sexual Activities Questionnaire (BESAQ), and the Endometriosis Health Profile Questionnaire (EHP-30).

**Results:**

The mean FSFI score in patients was 27.8 (SD5.4) versus 27.4 (SD6.8) in controls (p=0.858). A total FSFI score ≥26.55, indicating no sexual dysfunction was present in 70.6% of patients and 69.2% of controls (p=1.000). The mean BESAQ score in patients was 30.4 (18.5), compared to 38.3 (SD21.4) in controls (p=0.261), where lower scores denote better body image during intimate sessions. In the EHP-30, a statistically significant difference between patients and controls was found in all items on sexual intercourse. The subscale score of patients was 31.1 (SD26.2) versus 7.0 (SD11.1) in controls (p=<0.001), indicating better sexual functioning in controls.

**What is new?:**

The study showed that a history of menstrual outflow obstruction had a negative influence on several domains of sexual function, yet the patients total scores on sexual function remained in the normal range. The FSFI score of patients’ post-surgical treatment of obstructive congenital anomalies is similar to the control group.

## Introduction

Obstruction of menstrual blood flow due to congenital anomalies of Müllerian structures may lead to major problems such as menstrual disorders, recurrent vaginal infections and discharge, endometriosis, pain, fertility issues, and sexual dysfunction (Skinner and Quint, 2017; Deffarges et al., 2001; Zhang et al.,2017; Kim et al., 2021; Heinonen, 2001; Candiani et al., 1997). Surgical correction may be primarily aimed at resolving obstruction and pain relief but may also create a possibility for sexual intercourse and normal sexual function. In their publication, Masters and Johnson (1961) were one of the first to examine physiological sexual function after medical treatment for vaginal agenesis. They showed that similar sexual arousal occurred in patients with and without the anomaly (Masters and Johnson, 1961). Unfortunately, sexual function after surgical correction of obstructing Müllerian anomalies is under investigated in the scientific literature.

Deffarges et al. (2001) showed satisfactory sexual intercourse after the creation of a uterovaginal anastomosis in 12 women with uterine cervix atresia (Deffarges et al., 2001). Williams et al. (2014) evaluated 23 patients after resection of a transverse vaginal septum, of whom 61% had had menstrual obstruction. 47% of the 23 patients were sexually active and 35% of patients reported dyspareunia (Williams et al., 2014). A case report in 2019 described a woman with a high transverse vaginal septum (Sohail et al.,2019) who underwent a septum resection and use of an amnion graft. Post-procedure, she no longer has dyspareunia and successfully conceived (Sohail et al., 2019).

This paper aims to review sexual function and the prevalence of sexual dysfunction after surgical therapy for menstrual outflow obstruction due to congenital Müllerian anomalies. Our question to research is Does a history of surgically corrected menstrual obstruction result in more sexual dysfunction as compared to the general population?

## Materials and Methods

This long-term case-control study reports on secondary outcomes of a study focused on long- term abdominal pain and self-reported health state in women with menstrual obstruction with haematometra and/or haematocolpos due to congenital anomalies of the uterus, the uterine cervix or the vagina (Martens et al., 2024). IRDB status Medical ethical approval was obtained from the Committee on Research Involving Human Subjects region Nijmegen and Arnhem on February 26, 2015, under METC number 2014- 1186 and registered in the trial register under number: NL50025.091.14. The study was also approved by Radboudumc Board of Directors. Patients who had menstrual obstruction due to congenital anomalies and a surgical correction at our center between 1980 and 2013 were selected. Our center is a tertiary referral center, managing complex cases from this specific patient population. Patients had to be at least 18 years old and at least two years after the first operation at the start of this study. They gave informed consent to participate in the study before the questionnaires were distributed. The controls, recruited by a family doctor and a researcher, were women without known current gynaecological problems who filled out a questionnaire anonymously.

The ESHRE/ESGE consensus on the classification of female genital tract congenital anomalies was used (Grimbizis et al., 2013). Uterine, cervical, and vaginal anomalies were captured, providing consistency in reporting on the anomaly, categorisation and analysing patient group outcomes. Descriptive terminology as used in the American Society of Reproductive Medicine (ASRM) Müllerian anomalies classification 2021 would have been the alternative (Pfeifer et al., 2021).

Sexual function and experience were measured by the Female Sexual Function Index (FSFI) questionnaire, the Body Exposure during Sexual Activities Questionnaire (BESAQ), and the Endometriosis Health Profile Questionnaire (EHP30). The FSFI and EHP30 questionnaires were previously validated for the Dutch language. The EHP-30 questionnaire was validated in endometriosis patients and controls without known endometriosis (Van de Burgt et al., 2011; Ter Kuile et al., 2009; Wiegel et al., 2005; Rosen et al., 2000; Meston, 2003; Cash et al., 2004; Lowder et al., 2010; Jones et al., 2001). As the word ‘endometriosis’ was replaced by ‘abdominal pain’ for the questionnaire, it was not validated in this form.

The FSFI was validated in Dutch women with or without sexual complaints, but not specifically in women with obstructing Müllerian anomalies (Van de Burgt et al., 2011; Ter Kuile et al., 2009). The FSFI consists of 19 items measuring sexual desire, arousal, lubrication, orgasm, satisfaction, and genital pain that occurred during the previous four weeks with a lower score denoting a poorer sexual experience. For the FSFI, the cut-off value to discriminate between patients with and without sexual dysfunction was set at 26.55. Women with an FSFI score below 26.55 were previously defined as having sexual dysfunction (Ter Kuile et al., 2009; Wiegel et al., 2005; Rosen et al., 2000; Meston, 2003).

The BESAQ evaluates cognitions and behaviour during sexual intercourse. The 28-item questionnaire assesses avoidant and anxious body focus during sexual activity. No time interval was described. A lower score denotes better body image during intimate sessions. Scores of the 28 items range between 0 and 4. The total score ranges between 0 and 112. The BESAQ is calculated by averaging the item responses after reverse scoring specific items (Cash et al., 2004; Lowder et al., 2010). There is no cut-off point for normal versus abnormal available from the literature.

The EHP-30 consists of a core questionnaire and a modular questionnaire, with questions based on the patients’ experience in the previous four weeks. These questions related to the influence of abdominal pain on sexual relations. In this study, only the answers to 5 questions of the subscale Sexual intercourse in the modular questionnaire were analysed. The sum of scores on all items in a subscale divided by the maximum possible score of that subscale, multiplied by 100, leads to the calculated EHP-30 total subscale score equals. Higher scores indicate poorer sexual functioning (Van de Burgt et al., 2011; Jones et al., 2001).

### Statistical analyses

Statistical analyses were performed using the statistical software package IBM SPSS Statistics version 23 (IBM SPSS Statistics. IBM Corporation. 2015. Armonk, New York, United States). Medians and means were presented with corresponding interquartile range (IQR) and standard deviation (SD), respectively, and were rounded to one decimal. The chi-square test linear-by-linear Association or Fisher’s Exact Test was used as appropriate for the analysis of group differences in categorical data. The numerical continuous parameters were presented quantitatively. Analysis was performed with the use of the Mann-Whitney U test or the unpaired sample t-test. A p-value <0.05 was considered statistically significant.

## Results

38 of 78 patients who met the inclusion criteria gave informed consent and returned the questionnaire (48.7%). 54 women from the control group completed the questionnaire. (Table I) The baseline characteristics were similar. Median age at inclusion was 32 (26-34.5) in the patient group and 30 (24.3- 43) in the control (p=0.750). The median age at first surgery was 16 (range 15-18) and follow-up was for at least 6 years. In 24 (63.2%) patients the vagina had been obstructed (Table II). Patients 14- 28, classified as U3bC2V2, were diagnosed with OHVIRA (Obstructed Hemivagina and Ipsilateral Renal Anomaly) syndrome (Table I). 27 (71.1%) patients required multiple operations. 37 (97.4%) patients and 41 (75.9%) controls completed, or at least part of, the questionnaires on sexual function.

**Table I:** Study population: Müllerian anomalies and surgical interventions performed.

Patient numbers	Uterine anomaly	Cervical anomaly	Vaginal anomaly	Surgical intervention(s)
1-5	U0	C0	V3	Resection of hymen (n=4), Resection of (thin) septum and direct adaptation with dilator treatment (n=1)
6-12	U0	C0	V4	Resection of (thick) septum and direct adaptation with dilator treatment (n=6), Marsupialisation vagina with introitoplasty, and right heminephro-ureterectomy (n=1)
13	U1c	C4	V4	Hysterectomy and Davydov neovaginoplasty
14-28	U3b	C2	V2	Pull through technique and resection of septum (n=15)
29-36	U4a	C0	V0	Resection rudimentary horn and fallopian tube (n=8)
37	U4a	C4	V0	Resection rudimentary horn and fallopian tube and excision dysgenetic cervix
38	U4a	C0	V3	Resection rudimentary horn and fallopian tube and resection hymen

**Table II:** Characteristics of the study population.

Categories		n	Patients	n	Controls	p-value
Age (questionnaire), median (IQR), years		38	32 (26-34.5)	52	30 (24.3-43)	0.750^a^
BMI, median (IQR), kg/m^2^		38	23.9 (22-28)	51	23.0 (21-26)	0.216^a^
Smoking, n (%), yes		38	8 (21.1)	52	5 (9.6)	0.127^b^
Level of obstruction, n (%)	Vagina	38	24 (63.2)	-	-	-
	Hymen		4 (10.5)		-	
	Uterus		8 (21.1)		-	
	Cervix + Vagina		1 (2.6)		-	
	Uterus + Cervix		1 (2.6)		-	
Age (first complaints), median (IQR), years		32	15 (14-18)	-	-	-
Imaging performed for diagnosis, n (%)	Ultrasound	38	9 (23.7)	-	-	-
	Ultrasound + CT		2 (5.3)			
	Ultrasound + MRI		13 (34.2)			
	Ultrasound + CT + MRI		10 (26.3)			
Delay between complaints and diagnosis, median (IQR), months		29	6 (3-18)	-	-	-
Age (first operation), median (IQR), years		34	16 (15-18)	-	-	-
Multiple operations, n (%)		38	27 (71.1)	-	-	-
Pregnancies, median (IQR)		24	1 (0-2)	27	2 (1-3)	0.002^a^
Parity, median (IQR)		24	1 (0-1)	27	2 (1-3)	<0.001^a^

CT: computerized tomography scan; IQR: interquartile range; MRI: Magnetic Resonance Imaging; n: number; -: not applicable; p-value: level of test for statistical significant difference as calculated using a Mann-Whitney U test or b Chi-square test.

Table III reports the sexual function as measured by FSFI. 34 (89.5%) patients and 13 (24.1%) controls completed the FSFI questionnaire. The mean FSFI score in patients and controls were similar: 27.8 (SD5.4) versus 27.4 (SD6.8), respectively. The domains ‘Desire’ and ‘Lubrication’ showed statistically significant different results, with a better score in the patient group in the domain ‘Lubrication’. The controls scored better in the domain ‘Desire’. There was no significant difference between the percentage of patients or controls having a total FSFI score ≥26.55. 70.6% of patients with a surgically treated obstructive Müllerian anomaly had an FSFI total score ≥26.55 whereas in the control group, this was 69.2% (p=1.000).

**Table III:** FSFI scores (domains and total scores) in patients and controls.

	n	Patients	n	Controls	p-value
Desire	37	3.6 (1.3)	15	4.4 (1.3)	0.044^a^
Arousal	36	3.6 (1.1)	15	3.6 (1.6)	0.936^a^
Lubrication	37	4.9 (0.4)	15	4.3 (1.3)	0.013^a^
Orgasm	37	4.5 (0.6)	15	4.1 (1.3)	0.247^a^
Satisfaction	36	2.7 (1.0)	13	2.9 (1.3)	0.463^a^
Comfort/Pain	37	5.1 (2.1)	14	6.2 (1.6)	0.103^a^
Total score	34	27.8 (5.4)	13	27.4 (6.8)	0.858^a^
Total score ≥26.55 *	34	24 (70.6)	13	9 (69.2)	1.000^b^

Data are presented as a means with standard deviations per FSFI domain. n: number; p-value: level of statistical significance as measured with a Mann-Whitney U test or b Fisher’s Exact Test.

*The number of patients and controls with a total FSFI score ≥26.55 are given with the corresponding percent- ages.

Table IV reveals the results of the BESAQ. 32 (84.2%) patients and ten (18.5%) controls completed the BESAQ. The mean BESAQ score in the patient group was 30.4(SD18.5), compared to 38.3(SD21.4) in the control group (p=0.261).

**Table IV:** BESAQ scores (total scores and mean scores per item) in patients and controls.

	Patients (n=32)	Controls (n=10)	p-value
BESAQ total score	30.4 (18.5)	38.3 (21.4)	0.261
BESAQ mean score per item	1.1 (0.7)	1.4 (0.8)	0.261

Data are presented as means with standard deviations of the BESAQ scores. The scores of each item range between 0 and 4. The total score ranges between 0 and 112. Lower scores denote better body image.

BESAQ: Body Exposure during Sexual Activities Questionnaire; n : number ; p-value : level of statistical significance as measured with Mann-Whitney U test.

Table V reports the influence of abdominal pain on sexual intercourse during the last 4 weeks based on the modular questionnaire subscale Sexual intercourse of the EHP-30. 28(73.7%) patients and 32 (59.2%) controls completed the questions of the subscale. The subscale score of patients was 31.1 (SD26.2) compared to 7.0 (SD11.1) in controls (p=<0.001). This statistically significant difference between patients and controls was also found in all separate items of the subscale.

**Table V:** Scores on subscale ‘Sexual intercourse’ in the modular questionnaire of the EHP-30 in patient and control group.

	n	Patients	n	Controls	p-value
Subscale score, mean (SD)	28	31.1 (26.2)	32	7.0 (11.1)	<0.001
Items					
Experienced pain during or after intercourse?	34	1.4 (1.2)	41	0.3 (0.7)	<0.001
Felt worried about having intercourse because of the pain?	34	1.2 (1.2)	41	0.1 (0.3)	<0.001
Avoided intercourse because of the pain?	33	1.0 (1.3)	41	0.2 (0.7)	0.002
Felt guilty about not wanting to have intercourse?	33	1.1 (1.3)	41	0.2 (0.5)	<0.001
Felt frustrated because you cannot enjoy intercourse?	29	1.2 (1.3)	32	0.3 (0.7)	0.001

Data presented as means and standard deviations of the subscale score and all items within the subscale.

n: number; p-value: level of statistical significance as calculated with Mann-Whitney U test; SD: standard deviation.

The minimal-maximal group total subscale scores for patients and controls are 0-560, and 0-640, respectively.

The range of individual calculated EHP 30 total subscale scores is 0-100.

Table VI reports the effect of repair of different anatomic abnormalities on sexual function. FSFI: There was a mean total FSFI score below 26.55 in patients who underwent surgical neovagina creation by resection of vaginal septum, direct adaptation and dilator treatment or marsupialisation of the vagina with introitusplasty, which denotes sexual dysfunction in this group. BESAQ: The group of patients who underwent resection of hymen and patients who underwent surgical neovagina creation by resection of vaginal septum, direct adaptation and dilator treatment or marsupialisation of the vagina with introitusplasty had the lowest BESAQ total score, which denotes better body image during intimate sessions. EHP30: The group of patients who underwent resection of a rudimentary horn scored low on the adapted EHP30, which indicates better sexual functioning.

**Table VI:** FSFI total score, BESAQ total score, EHP30 subscale score in subgroups of patients.

	n	Patients who underwent resection hymen	n	Patients who underwent surgical neovagina creation by resection of vaginal septum, direct adaptation and dilator treatment	n	Patients who underwent resection of rudimentary horn	n	Patients who underwent vaginal pull through technique and resection of septum
FSFI total score	4	31.3 (3.2)	7	25.4 (8.3)	8	30.7 (1.9)	14	27.1 (3.8)
BESAQ total score	4	27.0 (22.4)	7	27.4 (16.2)	8	31.4 (13.6)	12	31.4 (13.8)
EHP30 Subscale score	3	26.7 (23.6)	5	44.0 (40.2)	8	17.5 (16.7)	11	36.8 (25.0)

Data presented as means and standard deviations.

BESAQ: Body Exposure during Sexual Activities Questionnaire; EHP30: Endometriose Health profile questionnaire; FSFI: Female Sexual Function Index questionnaire; n: number.

## Discussion

This study evaluated sexual function after surgically corrected congenital anomalies which caused menstrual outflow obstruction. The patient-reported overall sexual function does not seem to be negatively influenced by a history of menstrual obstruction. Not only the patients who have the need for a vaginal repair are prone to sexual dysfunction later in life.

More than two-thirds of patients (70.6%) in our study did not have sexual dysfunction after the resolution of menstrual obstruction, based on FSFI scores. Ter Kuile et al. (2009) found a total FSFI score of 16.7 (SD8.5) in Dutch women (N=234) with a sexual problem and 31.2 (SD3.9) in women without a sexual problem (N=108) (Ter Kuile et al., 2009). The mean FSFI total score in our patients was in the intermediate range, i.e. 27.8 (SD5.4), which is more in keeping with women without a sexual problem. The mean score in the control group was similar; 27.4 (SD 6.8). The FSFI does not discriminate between vaginal and clitoral orgasm which might however be a relevant issue in women after surgical correction of the vagina (Pastor et al., 2017).

Scores on the domain ‘Lubrication’ seemed to be better in the patients after menstrual obstruction compared to the control group. We hypothesise that this concerns a different underlying mechanism i.e. obstructing Müllerian anomalies such as transverse and longitudinal vaginal septa can cause persistent vaginal discharge, because the Müllerian epithelium may be altered in anomalies. Even after resolving the obstruction the tissues often produce more mucus (Kim et al., 2021; Heinonen, 2001; Candiani et al., 1997).

Sexual function is an important outcome measure after gynaecological reconstructive surgery, for example in patients with MRKH syndrome or with pelvic organ prolapse. Several other reports evaluated sexual function based on FSFI or BESAQ after surgical correction of genital anomalies (Lowder et al., 2010; Pastor et al., 2017; Weijenborg et al., 2019; Giannesi et al., 2005; Communal et al., 2003). There were no other studies on sexual function in an identical patient population to compare our results with. As all obstructive anomalies have been analysed as one group, the comparison to other studies with an individual approach is limited. Most other studies were on patients with MRKH syndrome, with or without menstrual obstruction. Pastor et al. (2017) analysed the sexual life of patients with MRKH syndrome after laparoscopic Vecchietti vaginoplasty. The FSFI total scores of patients were not statistically different from the age- matched control group, but they found a statistically significant poorer score among the patients in the domain ‘Lubrication’ (Pastor et al., 2017). Weijenborg et al. (2019) investigated the sexual function in 54 MRKH patients with a non-surgically or surgically created neovagina. MRKH women scored significantly lower on the FSFI domain Pain (P<0.05) (Weijenborg et al., 2019). Giannessi et al. (2005) studied the sexual function after Davydov neovaginaplasty in patients with MRKH syndrome compared to an age-matched control group consisting of women who accompanied the patients to the outpatient clinic. They found no statistically significant differences in the FSFI domains or total scores (Giannesi et al., 2005).

Pain, coping ability, psychological response, and patients’ body perceptions are important factors that may determine sexual function (Cash et al., 2004; Hecker and McGuire, 1977; Langer et al., 1990). The high number of missing variables in the BESAQ questionnaire, especially in the controls, hampered conclusions on such cognitions and behaviour during sexual intercourse. The data available showed little impairment in our patient group. As compared with patients who underwent reconstructive surgery for pelvic organ prolapse, the mean and total BESAQ scores in our patients were equal: 1.1 (SD0.7) versus 1.1 (SD0.7) and 30.4 (SD18.5) versus 30 (SD19) (Lowder et al., 2010). This may indicate that cognition and sexual behaviour are not influenced by the type of anomaly or surgery.

Based on the adapted EHP-30, patients experienced statistically significantly more pain during intercourse, had more worries about intercourse, avoided intercourse more frequently, and had more feelings of guilt and frustration about not wanting intercourse compared to the controls. This might be caused by changed anatomy, altered tissue, genesis of fibrosis, stenosis after surgery, and their behavioural and psychological consequences (Deffarges et al., 2001). Pain due to intercourse may lead to avoidance, worries and feelings of guilt. Moreover, an altered self-esteem due to gynaecological problems with clinical investigation and surgery during adolescence may play a role. The need for the postoperative use of a vaginal dilator will also have an influence. Endometriosis may be a result of menstrual obstruction, which can also lead to dyspareunia and sexual dysfunction. In their systematic review, Barbara et al. (2017) stated that patients with endometriosis have a higher risk of sexual dysfunction. Two-thirds of patients with endometriosis had some form of sexual dysfunction (dyspareunia, low satisfaction, lack of desire, low arousal, and/or orgasm difficulties). Not only pain during intercourse but also desire and the orgasmic capacity were negatively affected (Barbara et al., 2017).

The subgroup analysis of the effect on sexual function of different surgical strategies to repair different anatomic abnormalities showed that patients with Müllerian anomalies needing vaginal repair (excluding patients needing resection of hymen) had the lowest FSFI total scores. The BESAQ scores were similar in all subgroups. The results of the EHP-30 revealed the lowest scores after resection of a rudimentary horn, indicating a better sexual function.

The strength of this study is the long-term follow-up and relatively large study population with rare congenital anomalies. The response rate was relatively high in view of the long inclusion period. There was however a high number of missing data due to unanswered questions on sexual function, especially in the control group. It is assumed that some parts of the questionnaire, especially the FSFI and BESAQ questionnaire, covered too intimate/private topics, even though the questionnaire was returned anonymously. In the EHP-30 and FSFI, women could choose to fill out the options: don’t want to answer and not applicable or no sexual activity. There were missing data to these questions, and therefore we could not discriminate between patients who did not complete the questionnaire and who were not sexually active. Additionally, since each questionnaire has been validated for a purpose, comparing results was challenging. For example, there were statistically significant differences in the results from the EHP-30 subscale sexual intercourse, but these differences were not found in the scores from either FSFI or BESAQ. Thereby these findings may have limited relevance.

The main limitation of this study is that it is retrospective. Due to the low age at diagnosis in these conditions, it is not possible to prospectively assess changes from preoperative to postoperative scores. Interpretation of the results was hampered by the heterogeneity of the conditions, including the variation of Müllerian anomalies, the timing of diagnosis, the surgical approach, postoperative course (dilator use, repeat surgery), vaginal elasticity/capacity and comorbidities. Therefore, we performed a subgroup analysis on the effect of repair of different anatomic abnormalities on sexual function. This showed that not only Müllerian anomalies that needed vaginal repair interfered with sexual function. A future study may assess specific factors that may affect sexuality, such as confirmed endometriosis.

## Conclusions

This study revealed that history of menstrual obstruction has a negative influence on several domains of sexual function, but that the questionnaire’s total scores on sexual function were in the normal range. The FSFI score of patients with obstructive congenital anomalies is similar when compared to a control group. Not only those Müllerian anomalies that needed vaginal repair interfere with sexual function, since poorer scores (BESAQ) were also found in women with an obstructed uterine horn.
